# Supplementary figures and images for: Aberrant autophagosome formation occurs upon small molecule inhibition of ULK1 kinase activity
Source: Life Sci Alliance. 2020 Oct 27;3(12):e202000815. doi: 10.26508/lsa.202000815 (PMC7652397; doi:10.26508/lsa.202000815)

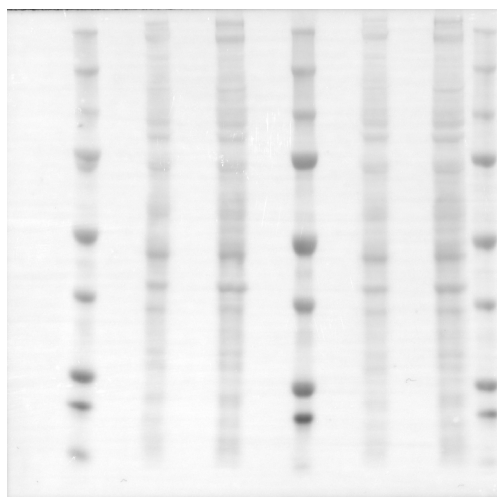

ponceau

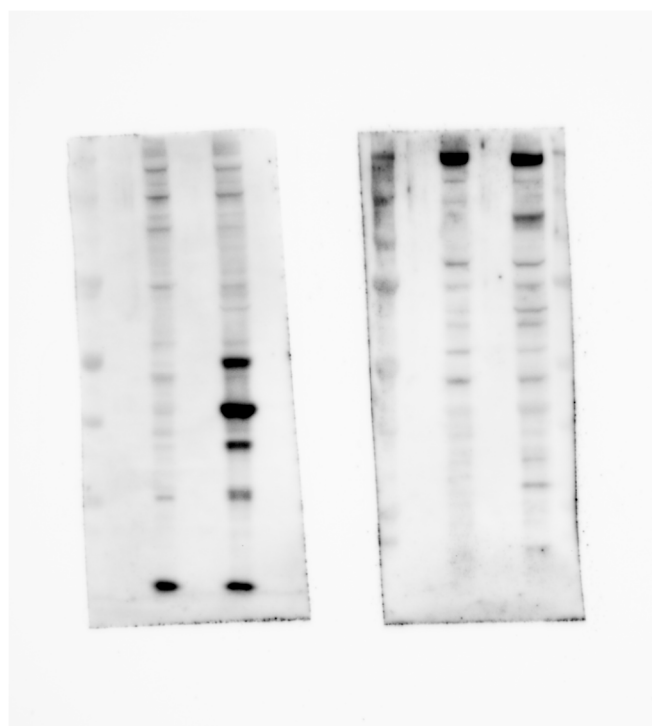

LC3

mCherry

Supplement: Supplementary file 1 [file LSA-2020-00815_SdataFS1.pdf]

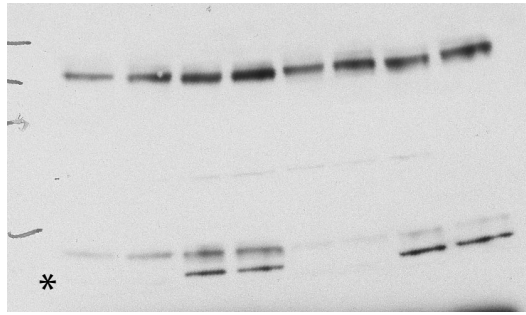

TOT ULK1

P Ser 318 ATG13

\* unspecific band

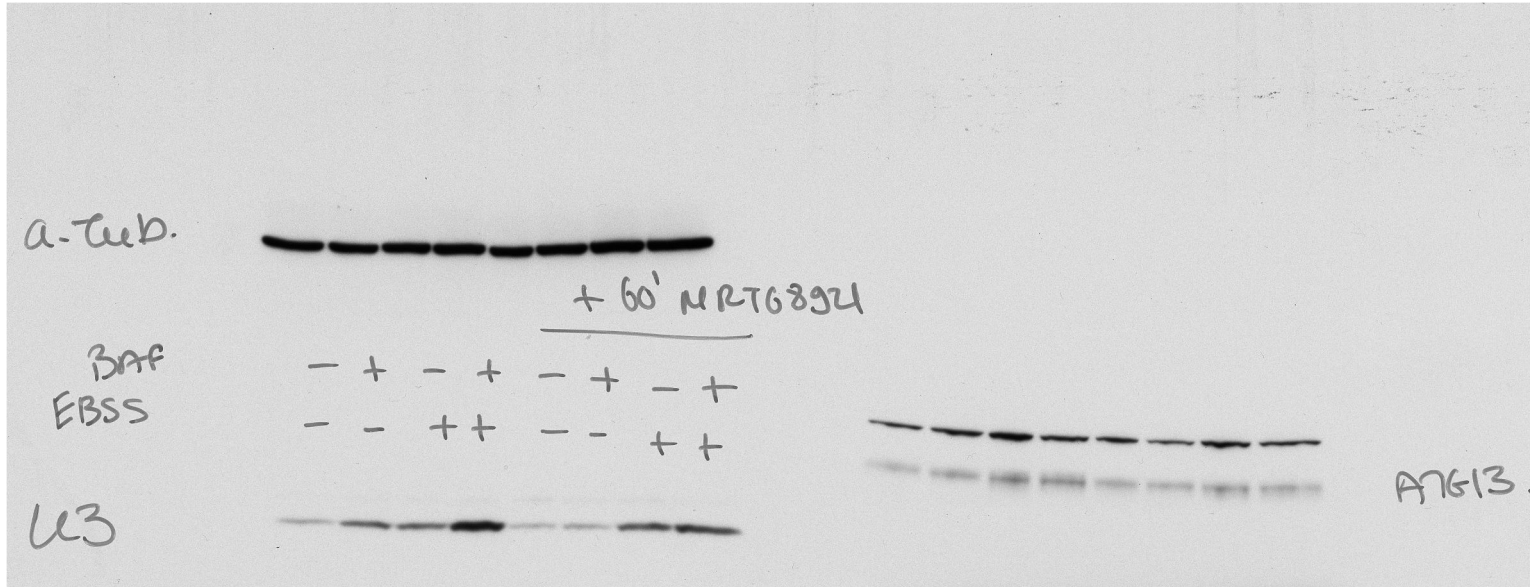

Supplement: Supplementary file 2 [file LSA-2020-00815_SdataF1.pdf]

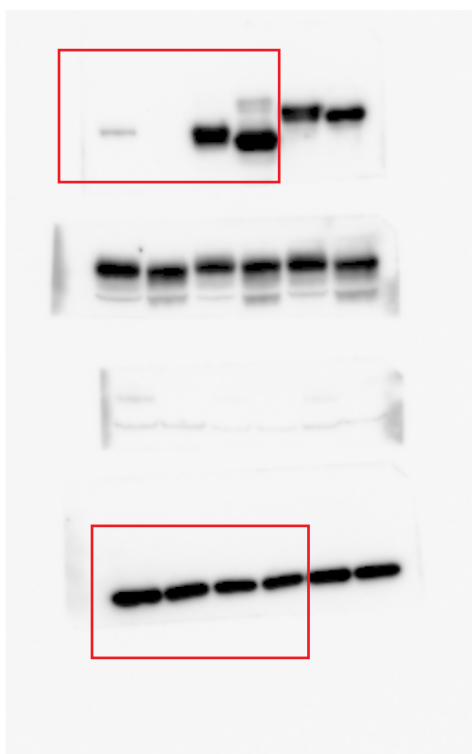

ULK1

GAPDH

Supplement: Supplementary file 5 [file LSA-2020-00815_SdataF2.pdf]

ATG13

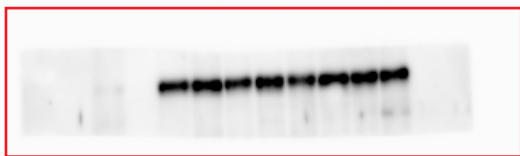

FIP200

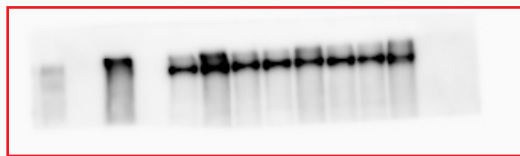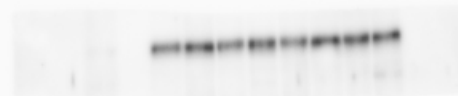

ULK1

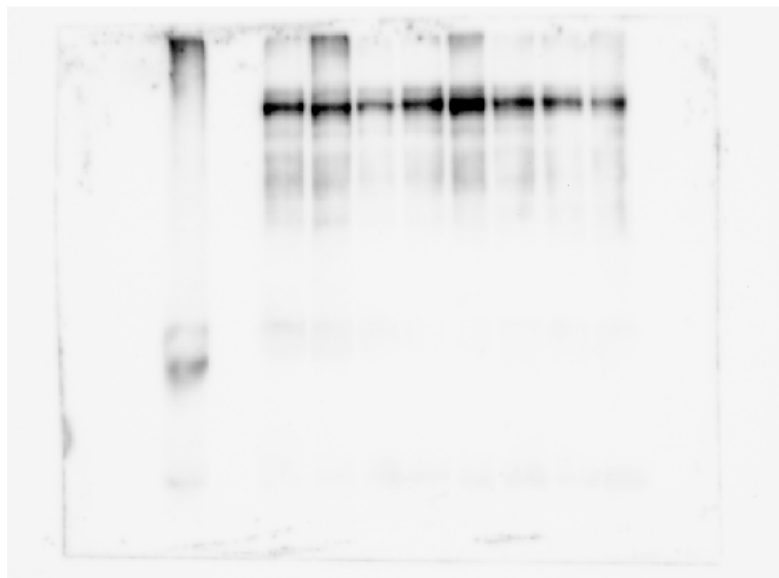

FIP200

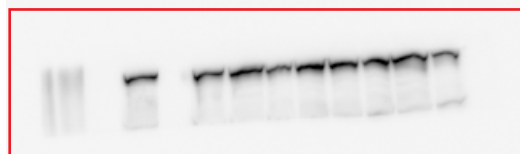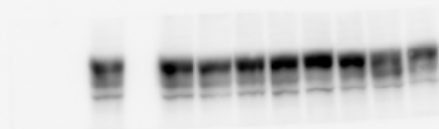

p Ser 318 ATG13

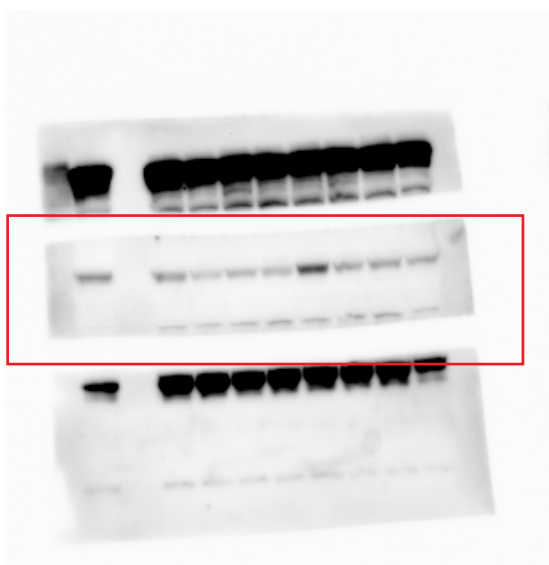

ATG13

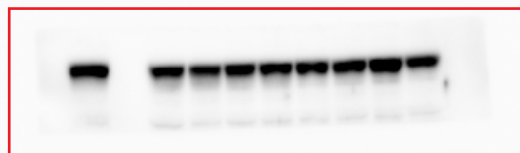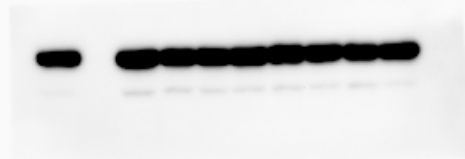

ULK1

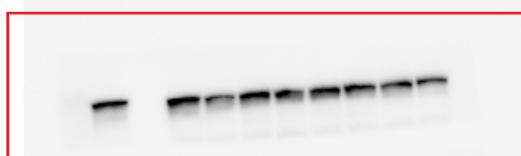

GAPDH

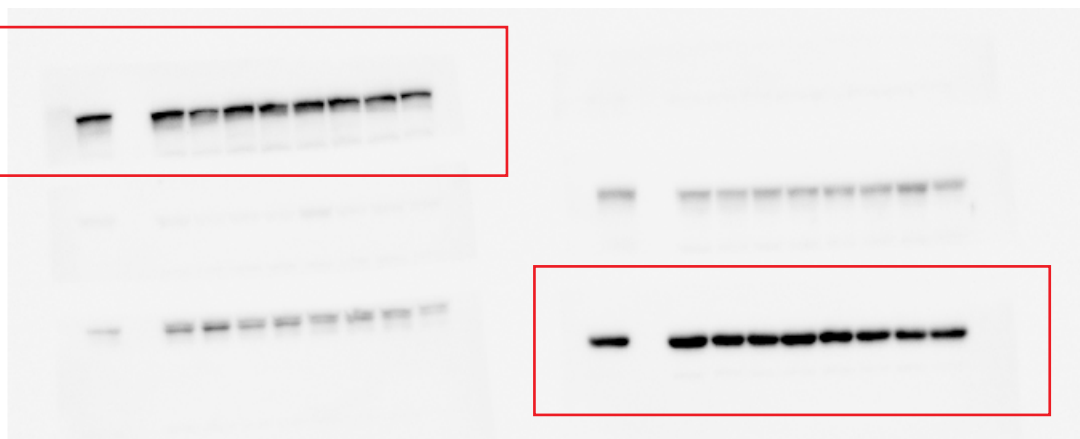

Supplement: Supplementary file 7 [file LSA-2020-00815_SdataFS3.pdf]
