# Supplementary material for: Aberrant autophagosome formation occurs upon small molecule inhibition of ULK1 kinase activity
Source: Life Sci Alliance. 2020 Oct 27;3(12):e202000815. doi: 10.26508/lsa.202000815 (PMC7652397; doi:10.26508/lsa.202000815)
Supplement: Supplementary file 6 [file LSA-2020-00815_SdataF3.pdf]

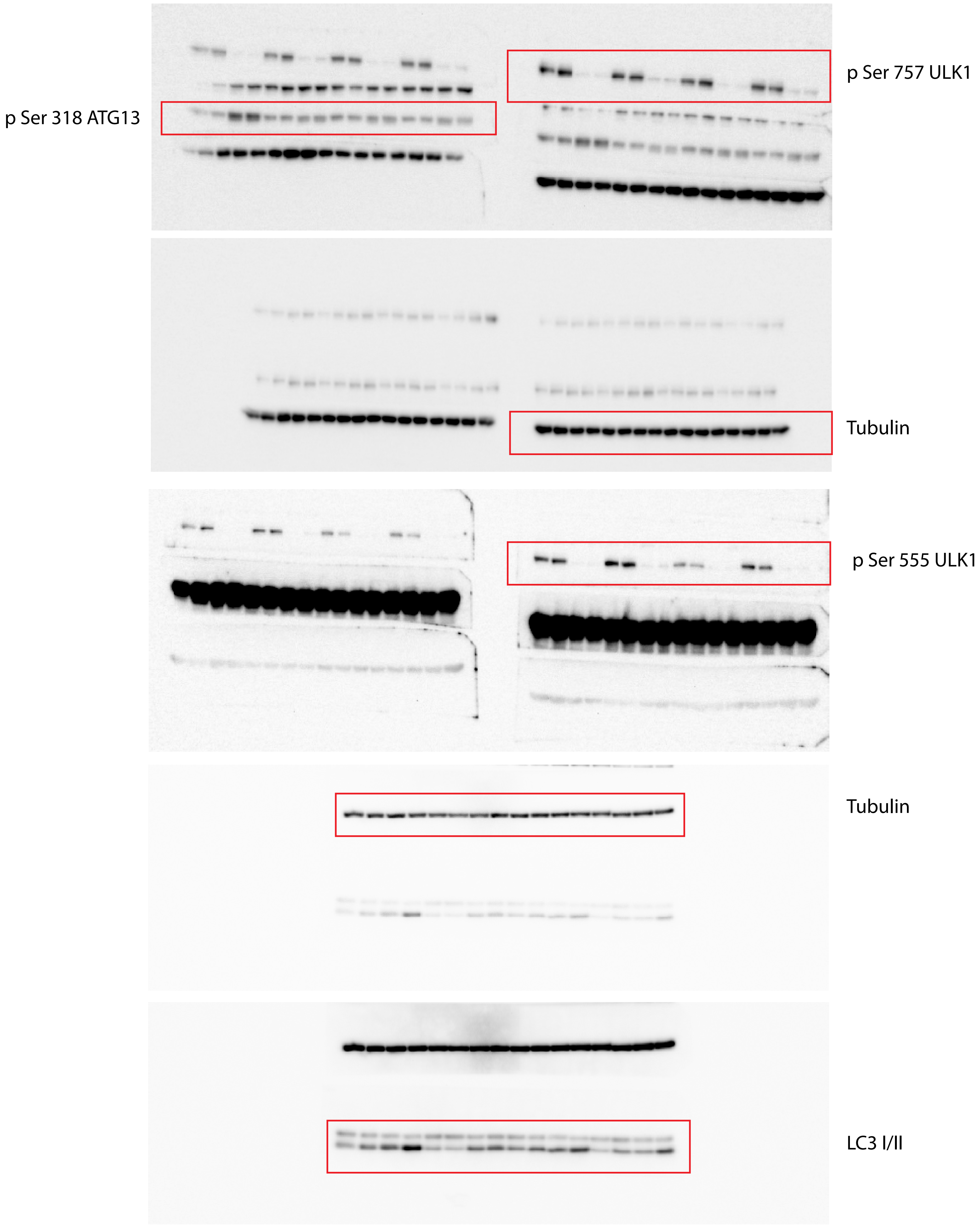

p Ser 757 ULK1
p Ser 318 ATG13
Tubulin
p Ser 555 ULK1
Tubulin
LC3 I/II

ULK1

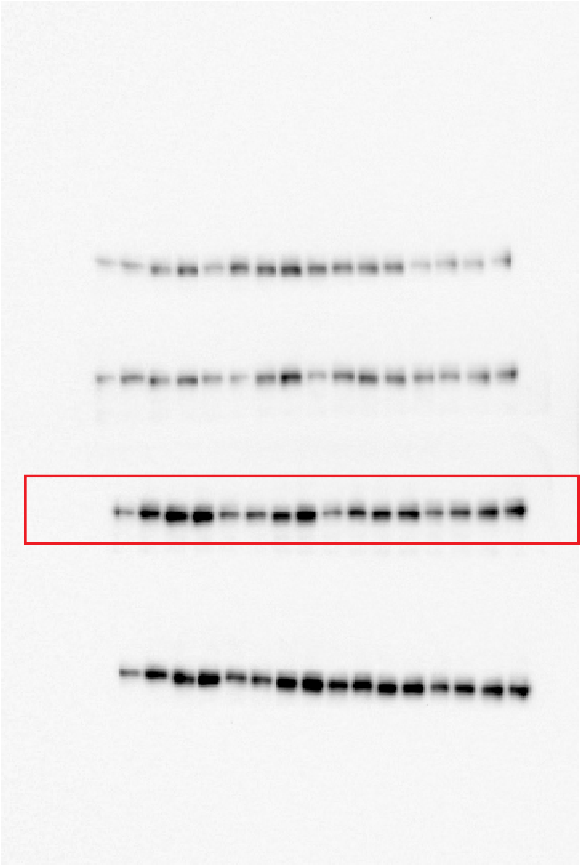

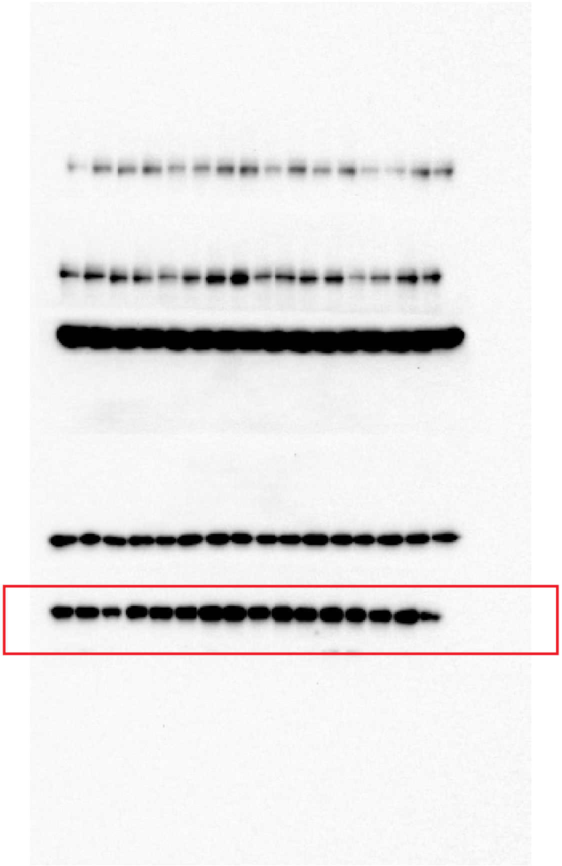

ATG13

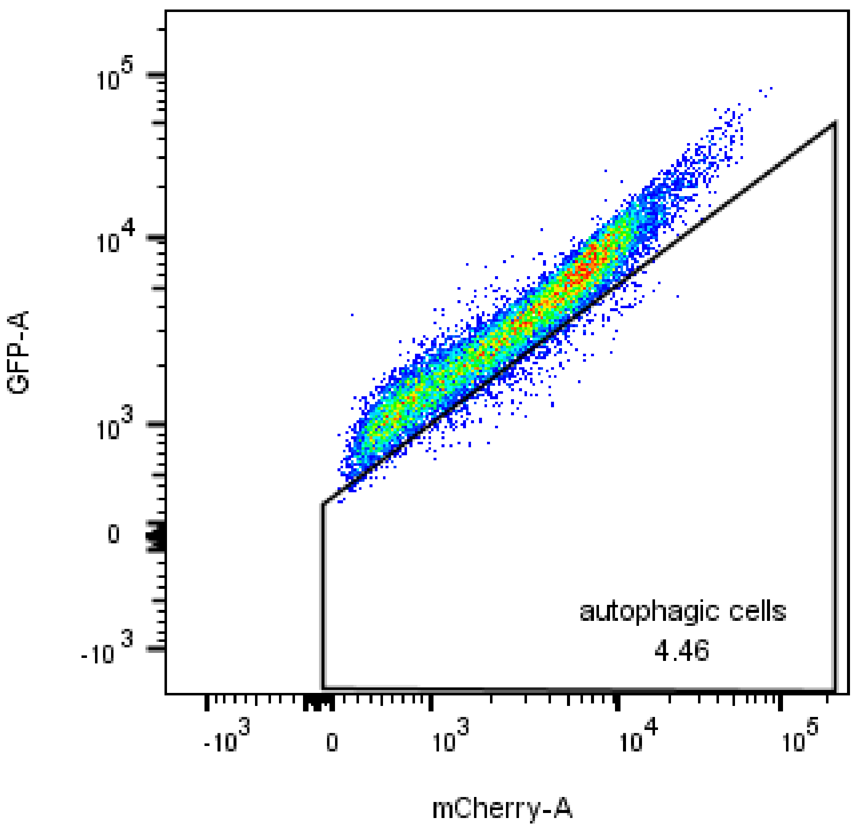

Specimen_001_ctr_006.fcs
Single Cells
16066

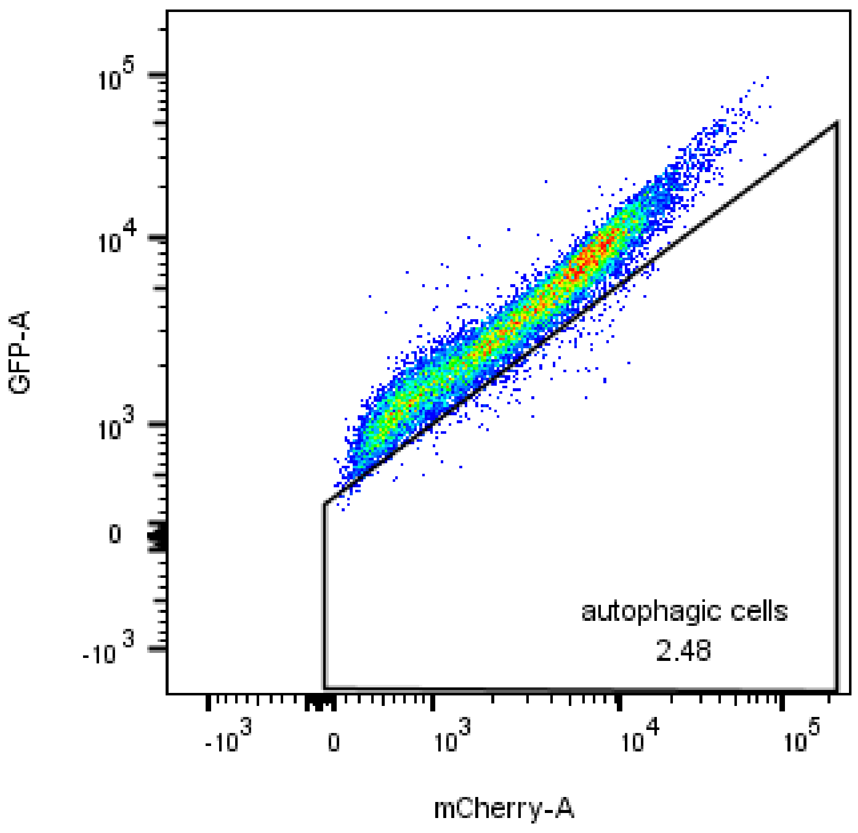

Specimen_001_ctr baf_007.fcs
Single Cells
16315

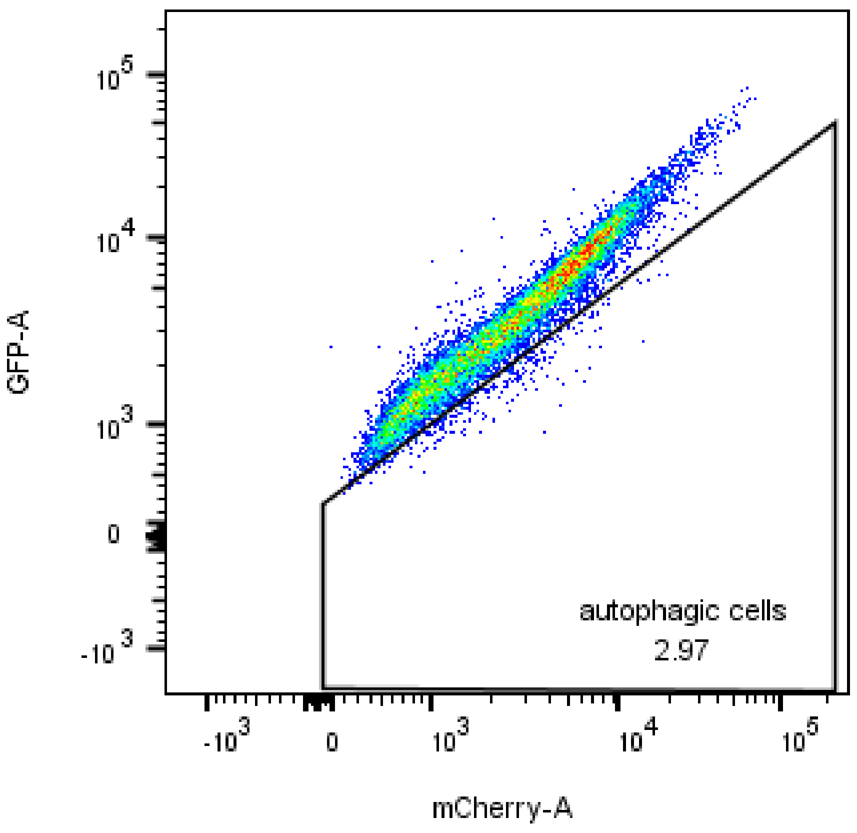

Specimen_001_ctr mrt_008.fcs
Single Cells
14505

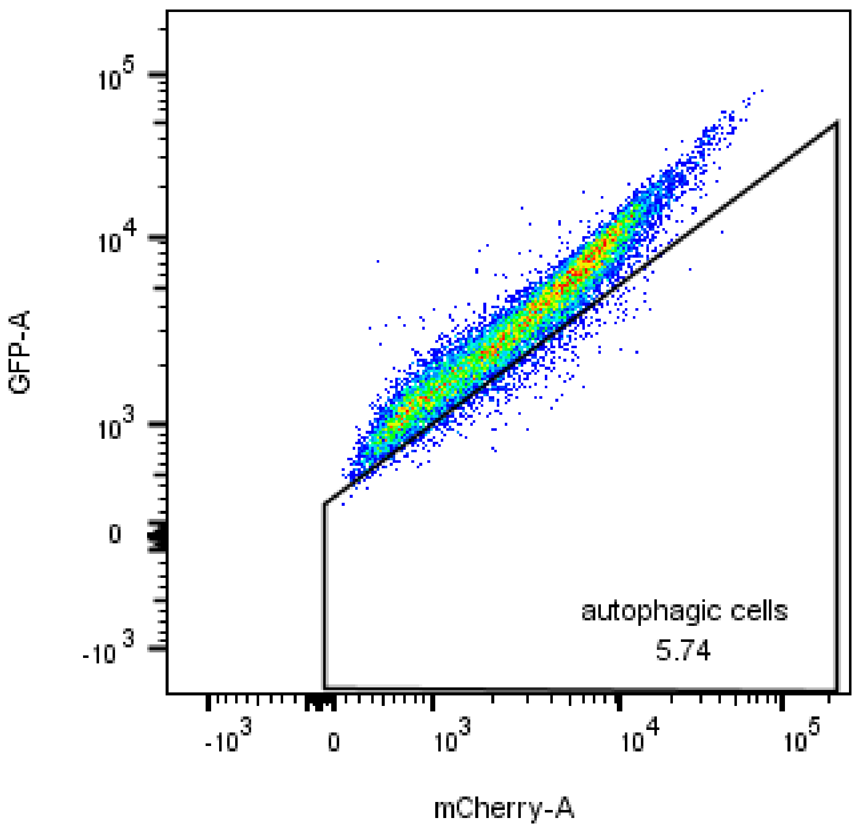

Specimen_001_ctr sbi_009.fcs
Single Cells
14524

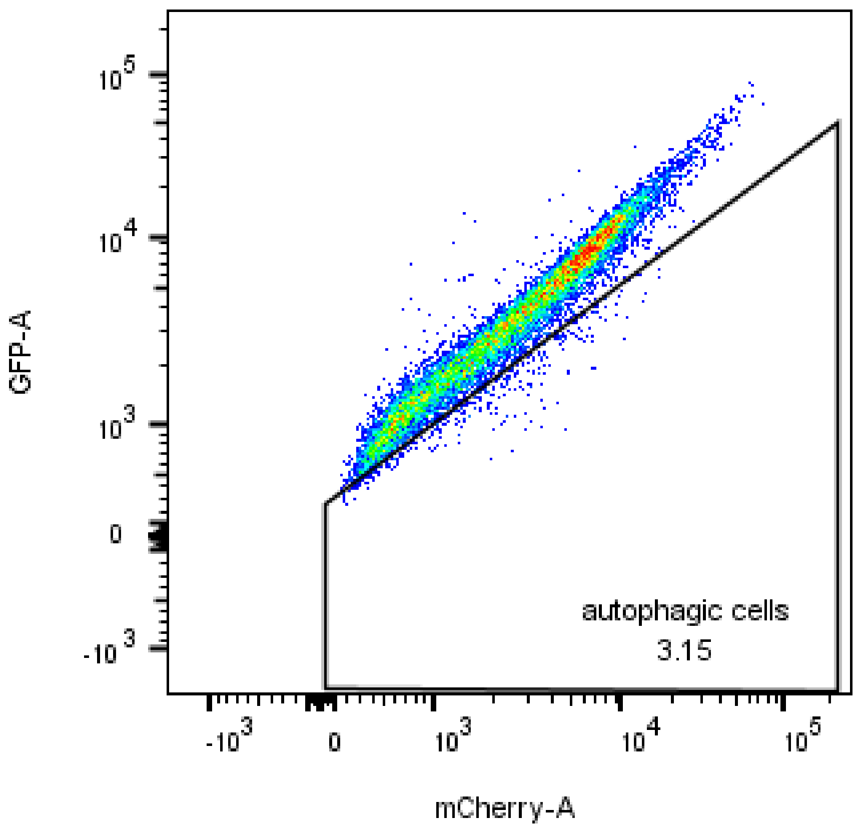

Specimen_001_ctr 101_010.fcs
Single Cells
14430

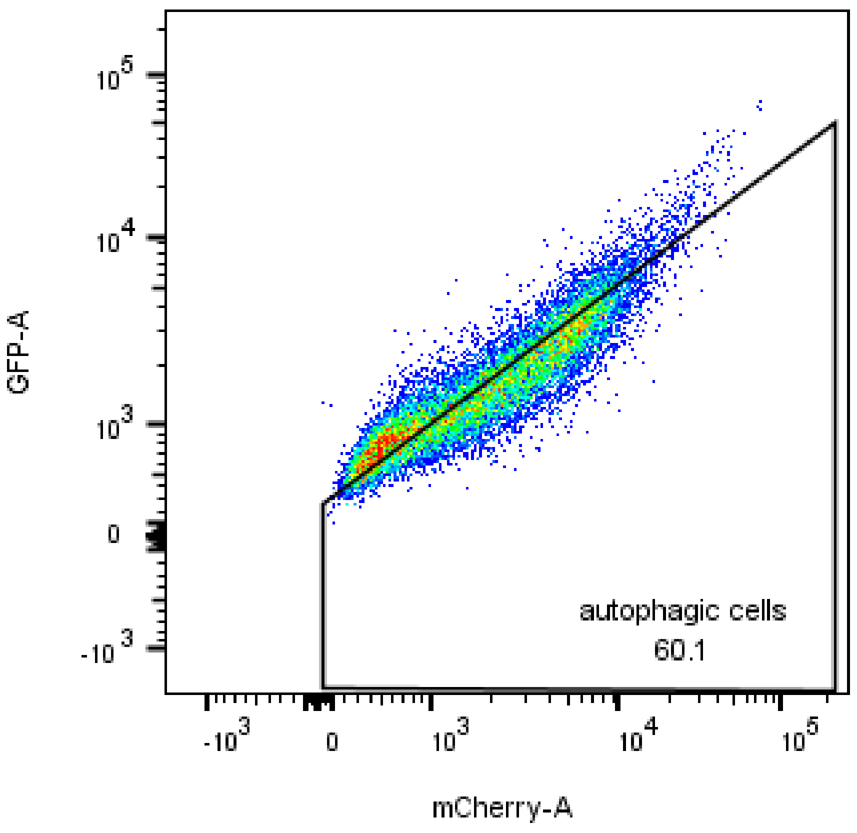

Specimen_001_ebss_005.fcs
Single Cells
15767

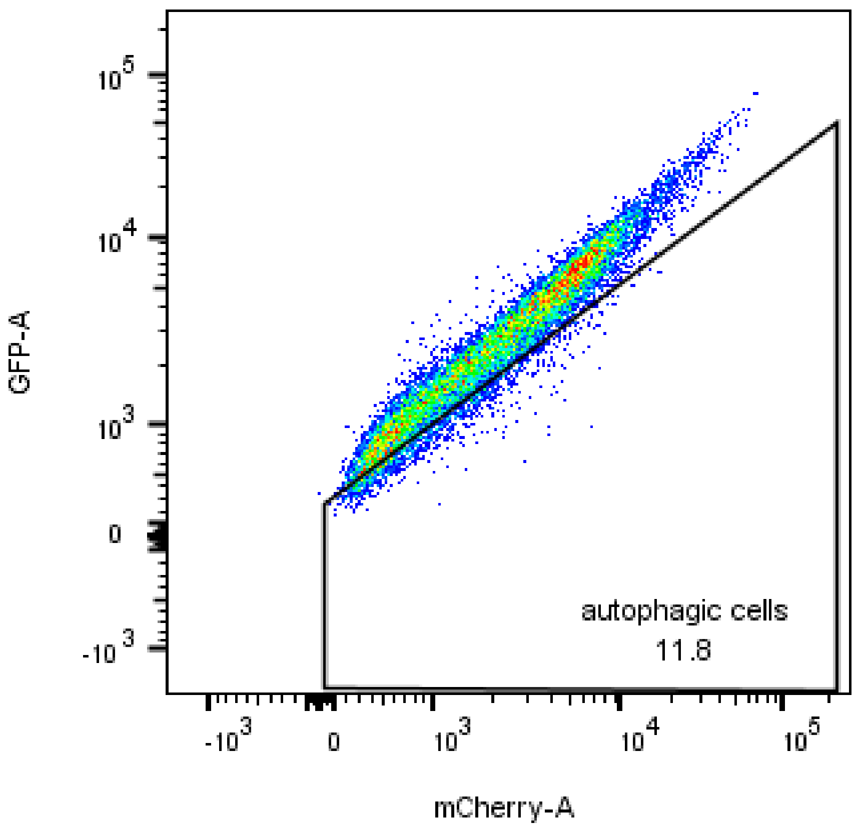

Specimen_001_ebss baf_004.fcs
Single Cells
14756

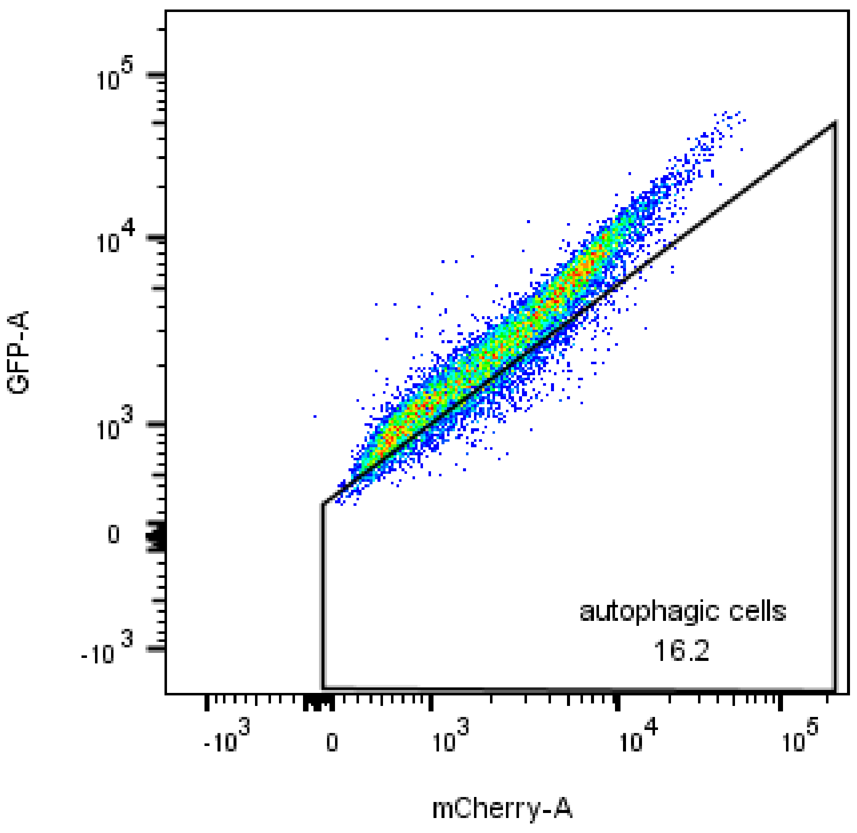

Specimen_001_ebss mrt_003.fcs
Single Cells
12011

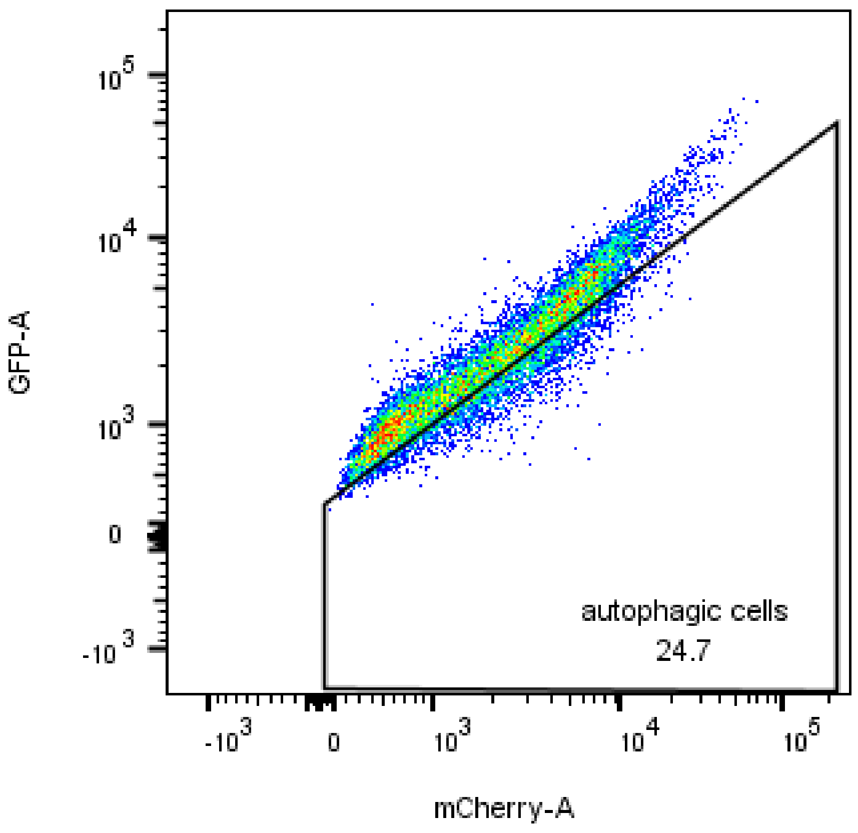

Specimen_001_ebss sbi_001.fcs
Single Cells
13079

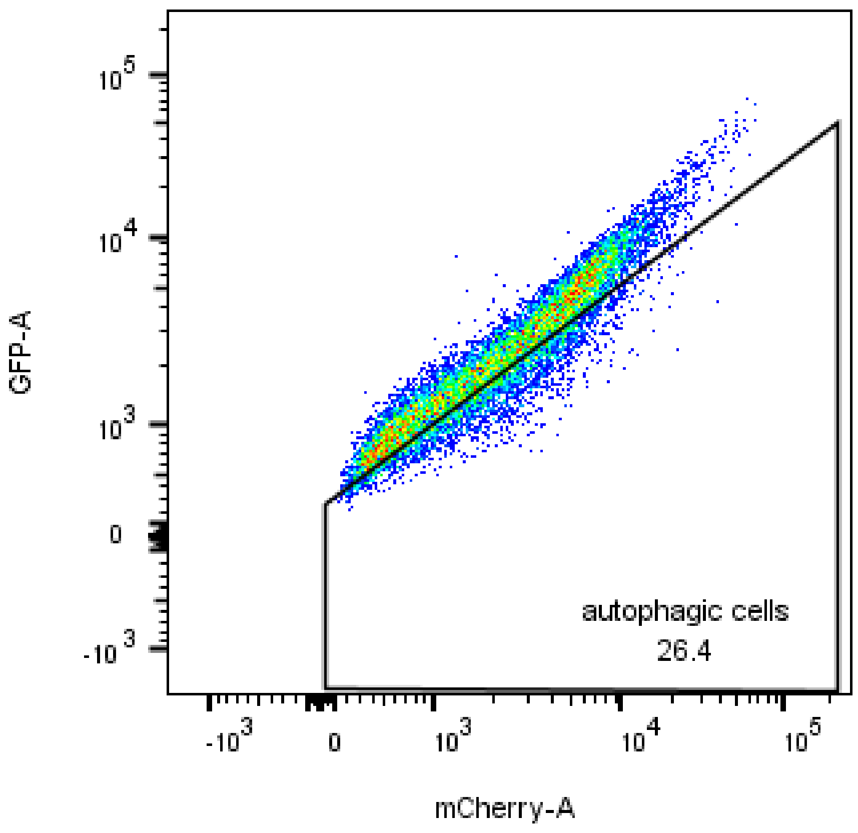

Specimen_001_ebss 101_002.fcs
Single Cells
14586
